# Transcriptional and Post-transcriptional Gene Regulation by Long Non-coding RNA

**DOI:** 10.1016/j.gpb.2016.12.005

**Published:** 2017-05-19

**Authors:** Iain M. Dykes, Costanza Emanueli

**Affiliations:** 1 School of Clinical Sciences, University of Bristol, Bristol BS2 8HW, United Kingdom; 2 National Heart and Lung Institute, Imperial College London, London SW7 2AZ, United Kingdom

**Keywords:** Long non-coding RNA, MicroRNA, Transcriptional regulation, Epigenetics, Post-transcriptional regulation

## Abstract

Advances in genomics technology over recent years have led to the surprising discovery that the genome is far more pervasively transcribed than was previously appreciated. Much of the newly-discovered transcriptome appears to represent **long non-coding RNA** (lncRNA), a heterogeneous group of largely uncharacterised transcripts. Understanding the biological function of these molecules represents a major challenge and in this review we discuss some of the progress made to date. One major theme of lncRNA biology seems to be the existence of a network of interactions with **microRNA** (miRNA) pathways. lncRNA has been shown to act as both a source and an inhibitory regulator of miRNA. At the transcriptional level, a model is emerging whereby lncRNA bridges DNA and protein by binding to chromatin and serving as a scaffold for modifying protein complexes. Such a mechanism can bridge promoters to enhancers or enhancer-like non-coding genes by regulating chromatin looping, as well as conferring specificity on histone modifying complexes by directing them to specific loci.

## The emerging field of long non-coding RNA

Recent advances in sequencing technologies have demonstrated that far more of the genome is transcribed than was previously appreciated. There has been an explosion in the number of described non-coding genes and with it a corresponding surge in interest in this emerging field. The term “dark matter” was coined to describe the large number of previously-overlooked transcripts of uncertain function revealed by such work [1], [2] indicating the existence of a large non-coding transcriptome far exceeding that of the more familiar coding genes. The rate of discovery has far outpaced our ability to functionally characterise these transcripts and the vast majority have no known function. Despite this, the importance of the dark matter is demonstrated by genome-wide association studies (GWAS) which have indicated that long non-coding RNA (lncRNA) genes are enriched for trait or disease-linked polymorphisms [3], [4] and, indeed, over 90% of all GWAS hits lie outside of known coding genes [5]. This review will be focussed on efforts to understand the biological function of lncRNA. Of necessity, we will be focussing on a small number of the best-known genes, but throughout we will discuss to what extent these may represent widespread mechanisms.

### The diversity of lncRNA

A large proportion of the genome is transcribed into RNA that lacks protein coding information and is never translated [6], [7], [8]. These non-coding RNAs (ncRNAs) are generally divided into long and short groupings using an arbitrary threshold of 200 nucleotides (nt). Short ncRNAs include the relatively well-known microRNAs (miRNAs) [9], [10], as well as small-interfering RNAs (siRNAs) and P-element-induced wimpy testis (PIWI)-interacting RNA (piRNAs) [11], which generally act to negatively regulate gene expression. In contrast, the long ncRNA (lncRNAs) are a large, heterogeneous group of ncRNAs of largely-unknown function. lncRNAs share many features with coding transcripts, such as the presence of epigenetic marks indicating differential expression [12], the presence of introns, and the existence of splice variants. Many but not all lncRNAs are polyadenylated, and there is evidence indicating that many lncRNAs exist in both polyadenylated and non-polyadenylated forms (termed bimorphic) [3]. The broad term lncRNA includes many different types of RNA, exhibiting a range of genomic structures and relationships to the coding transcriptome. Some are pseudogenes, copies of coding genes harbouring mutations rendering them non-coding [13]. Many lncRNAs overlap coding genes, and indeed, one estimate suggests that 20% of human transcripts exist as sense-antisense pairs [14]. These transcripts may overlap the entire gene or only a part of it, and non-coding transcripts may originate from either the sense or antisense strand [15], [16]. Many lncRNAs are described as intergenic (meaning that they do not lie within or overlap coding genes) and are sometimes known as long intergenic ncRNAs (lincRNAs) [3]. We now know that transcripts do not have to be linear, with the discovery that circular RNA (circRNA) is a common form of transcribed RNA [17], [18]. Although many circRNAs are transcribed from coding regions, these transcripts are believed to be non-coding.

With the discovery of all of these forms of RNA, the genome can no longer be thought of as a linear array of distinct transcriptional units, but rather is “*an amazingly complex landscape of interlacing and overlapping transcripts, not only on opposite strands, but also on the same strand, so that there is often no clear distinction between splice variants and overlapping and neighbouring genes*” [19].

### How much of the genome is transcribed?

Tiling microarrays, in which arrays are made of probes at short intervals designed to span the entire genome, were the first to suggest nearly pervasive transcription of the entire genome [1]. One study by Affymetrix on our smallest chromosomes, Chr.21 and Chr.22, has indicated that 94% of probes detect transcripts outside of known exons [6], while another study suggests that 49% of transcribed nucleotides are outside of any annotated gene [20]. The development of massively parallel sequencing technologies, commonly referred to as RNA sequencing (RNA-seq), has offered many improvements over microarray-based technology in terms of reproducibility, sensitivity, coverage, and accuracy in mapping homologous sequences [21]. The Encyclopaedia of DNA Elements (ENCODE) project has utilised RNA-seq of 15 human cell lines to demonstrate that 74.7% of the genome is transcribed [7], while a similar experiment utilising RNA-seq of samples derived from 23 different human tissue types indicated that 85% of the genome is transcribed [3]. The Functional Annotation of the Mammalian Genome (FANTOM) project has revealed a similar level of pervasive transcription with 63% of the genome shown to be transcribed by using the cap analysis of gene expression (CAGE) to profile transcription initiation sites within the genome [8]. A related method is to predict the location of transcribed regions of the genome based on the pattern of histone modification. Actively-transcribed genes are marked by trimethylation of lysine 4 on histone H3 (H3K4me3) at their promoters and H3K36me3 along the length of the transcribed regions. These so-called K4-K36 regions can be used in genome-wide chromatin precipitation assays to predict transcripts [12].

Thus it seems that the majority of our DNA is transcribed into RNA at some point during development. However, much of the previously-unappreciated ncRNA exists at a very low level. Most of the RNA in a cell is rRNA and tRNA, whereas mRNA makes up 3%–7% of the total by mass. lncRNA makes up 0.03%–0.20% [22], thus being 15–230 times less abundant in the cell than coding transcripts.

## Functions of lncRNA

While the vast majority of lncRNAs do not yet have any known function, we are beginning to understand the functions of a small number of characterised lncRNAs. lncRNAs can act at many different levels of gene expression and their functions are highly diverse. This diversity reflects the versatility of RNA itself: through folding into a variety of secondary structures, RNA can bind to a large number of substrates in a highly-specific manner [23]. In addition, without the need for translation, ncRNA expression is highly dynamic and can be rapidly up- or down-regulated to modulate gene expression [23]. Here, we will review some of the major described functions and highlight what appear to be general principles of lncRNA biology. For a more detailed discussion on this subject, the reader is directed to several of the many excellent reviews published [23], [24], [25], [26].

### Post-transcriptional regulation by lncRNA

#### lncRNA as a source of miRNA

miRNA is produced from primary transcript (pri-miRNA) by two processing stages. Drosha and DiGeorge syndrome chromosomal region 8 (DGCR8) cut the pri-miRNA while it is in the nucleus into a precursor (pre-miRNA) of ∼60 nt. The pre-miRNA is then exported to the cytoplasm where it is processed by a second enzyme complex, Dicer/TAR RNA protein (TRBP), to produce the mature miRNA of 20–23 nt [10], [27]. Most pri-miRNAs are generally greater than 1 kb in length [27], and therefore may be regarded as a form of lncRNA. There are two major sources of pri-miRNAs in the genome: those that are embedded within another gene and whose expression is thus normally, but not always, linked to the expression of the parent transcript; and those that are transcribed independently from what had previously been considered to be intergenic regions. The latter group is transcribed from miRNA genes, which contain promoters that regulate their transcription by RNA polymerase II (RNAPII) in a manner similar to mRNA [28].

**Figure 1:**
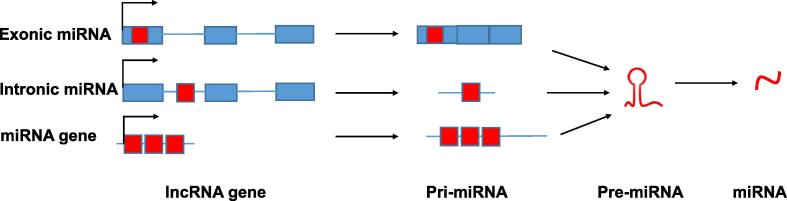
**lncRNA as a source of miRNA** Many lncRNA genes contain embedded miRNA sequences (red boxes), which may be located within either an exon (blue box) or an intron (line) of the gene. Furthermore, miRNAs are encoded by independent transcriptional units and often occur in clusters within the genome. The three sources result in very different types of primary transcript but the pathways converge at the level of pre-miRNA structure. lncRNA, long non-coding RNA; miRNA, microRNA; pri-miRNA, primary miRNA; pre-miRNA, precursor miRNA.

Approximately 50% of miRNAs are produced from non-coding transcripts [29] (Figure 1). Interestingly, in common with those embedded in coding genes, many miRNAs within non-coding genes are also located within introns [28]. Such a genomic organisation suggests that the host lncRNA does not simply act as a pri-miRNA but may have other additional roles encoded by the exons. Examples of lncRNAs harbouring intronic miRNAs include *DLEU2*, which is the host gene of the tumour suppressor miR-15a/16.1 cluster located within its third intron [30], [31]. These miRNAs are frequently down-regulated in leukaemia. Interestingly, in adult chronic lymphocytic leukaemia, the expression of miR-15a/16.1 seems to be regulated by the host gene promoter, which is bound by the transcription factors (TFs) MYC [31] and paired box 5 (PAX5; previously also known as B-cell-specific activator protein, BSAP) [32], while in childhood acute myeloid leukaemia, data from methylation assays indicate that this miRNA cluster is regulated independently of its host gene [33]. A second example is the tumour suppressor miR-31 that is down-regulated in breast cancer. *MIR-31* gene is embedded within an intron of the lncRNA *LOC554202* and its transcription is regulated by the methylation state of the host gene promoter [34].

A minority of lncRNA-embedded miRNAs reside not within introns but within an exon of the spliced lncRNA [28], [35]. Many such lncRNAs are named for the miRNA which they encode. For example, the lncRNA *MIR155 host gene* (*MIR155HG*; formerly known as B-cell integration cluster, *BIC*) harbours an exonic miRNA, miR-155, and this region of the lncRNA shows the strongest cross-species conservation [36], [37]. Similarly, *MIR22HG* encodes miR-22 within its second exon [35], while *MIR17HG* harbours a cluster of six miRNAs within its second exon [38].

One of the first lncRNAs to be discovered and perhaps the most studied, *H19*, contains miR-675 embedded within its first intron [39]. Although the *H19* transcript is widely expressed in the mouse embryo, miR-675 expression is limited to the placenta [40]. This indicates that processing of the *H19* transcript to release miR-675 is inhibited, which would seem to be mediated by binding of a RBP, human antigen R (HuR), to a site upstream of miR-675, thus blocking Drosha processing of the primary transcript [40]. Furthermore, the disparity between *H19* and miR-675 expression suggests that *H19* may not simply function as a pri-miRNA but may have additional functions. This hypothesis is supported by the discovery of additional *H19* functions (discussed below).

#### lncRNA as a negative regulator of miRNA

**Figure 2:**
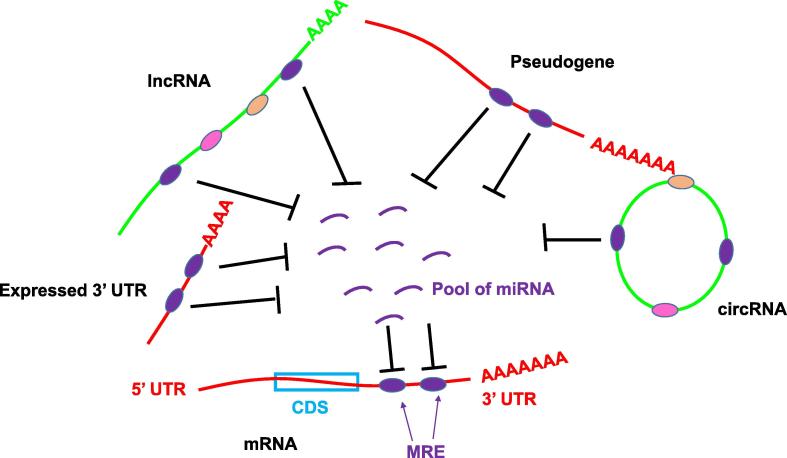
**The ceRNA hypothesis** mRNA contains MREs (ovals), which are normally located within the 3′UTR. miRNA binding to the identical MREs may be present in a number of ncRNA species, including pseudogenes, circRNAs, other forms of lncRNA, and independently-transcribed mRNA 3′UTRs. All of these RNAs could potentially compete for a limited pool of miRNA, thus positively regulating gene expression. lncRNA and circRNA may carry MREs for multiple miRNAs (indicated by differently coloured ovals). MRE, miRNA response element; UTR, untranslated region; miRNA, microRNA; lncRNA, long non-coding RNA; circRNA, circular RNA; CDS, coding sequence; ceRNA, competing endogenous RNA.

miRNAs are negative regulators of gene expression. Transcripts are targeted through binding of a short 7-nt seed sequence within the miRNA to an miRNA response element (MRE). MREs are short and binding does not have to be perfectly complementary [41], which makes predicting miRNA targets difficult. Computational predictions suggest that, potentially, a single miRNA may target hundreds of transcripts [41]. However, the number of target genes that are physiologically relevant targets of a given miRNA is often much lower [42]. There seems to be a disconnection between the number of predicted targets and the number of actual targets. Given the promiscuity of miRNA seed sequences, it is perhaps unsurprising that many lncRNAs contain predicted miRNA binding sites. This raises an interesting possibility that the function of many lncRNAs may be to regulate gene expression by sequestering miRNAs, thus limiting their concentration within the cell and thereby reducing the pool of available miRNA in the cell. In this way, the lncRNA acts as a negative regulator of miRNA function and, by extension, a positive regulator of gene expression. This is known as the “competing endogenous RNA (ceRNA)” hypothesis [43] (Figure 2).

Examples of this type of interaction include the intergenic *lincRNA-ROR*, which inhibits miR-145 in pluripotent embryonic stem cells (ESCs) [44]. *lincRNA-ROR* expression is activated by pluripotent TFs such as NANOG, SOX2, and OCT4 and genes encoding these TFs are targeted by miR-145. Therefore, this lncRNA creates a feedback loop within the pluripotent gene network [44]. *OCT4* expression is up-regulated in many cancers including hepatocellular carcinoma [45], and thus in these cells miR-145 acts as a tumour suppressor. Interestingly, a non-coding pseudogene of *OCT4* called *OCT4-pg4* is co-expressed with *OCT4* and appears to serve as an endogenous competitor of *OCT4*, protecting *OCT4* from miR-145-mediated degradation [46]. Pseudogenes are copies of coding genes that arise through DNA duplication followed by the accumulation of mutations in one copy, rendering the gene non-coding. Despite this, many pseudogenes are expressed as lncRNAs. Clearly, a non-coding transcript that shares a high degree of homology with a coding gene is likely to share many of its MREs and therefore pseudogenes are good candidates to act as ceRNAs. Indeed, this seems to be the case [13], [47], [48]. Examples of such lncRNAs include a pseudogene homologous to the gene encoding tumour suppressor phosphatase and tensin homologue (*PTEN*), which contains multiple MREs within the 3′UTR shared with the coding gene [48], as well as pseudogenes homologous to the 3′UTRs of the genes encoding tumour suppressor candidate 2 (*TUSC2*) [49] and forkhead box protein O1 (*FOXO1*) [50].

Some researchers remain sceptical about how widespread this mechanism may be, arguing that the low levels of expression of most lncRNAs relative to mRNAs means that alterations in lncRNA levels will not have more than a minor effect on miRNA availability, and so will be ineffective as a competitor [51]. Indeed, many studies on ceRNAs rely on ectopic overexpression of the ceRNA at artificially-elevated levels [49], [50]. Thomson and Dinger argue that while the ceRNA hypothesis is attractive because it could provide an explanation of the functions for so many uncharacterised transcripts, its biological relevance may be limited [51]. mRNA would be a more effective ceRNA for this reason and, interestingly, the ceRNA hypothesis is not limited to lncRNA. Salmena et al. [43] suggest that mRNAs could also function, in part, to regulate the expression of other mRNAs through a similar mechanism. If true, the mRNA of some coding genes may have a protein-independent non-coding function [43], in effect acting as a lncRNA. This is supported by the observation that the 3′UTRs of over 1500 human mRNAs are expressed independently of the coding part of the same transcript [52]. Furthermore, in about half of such genes examined in mice, the expression pattern of the independent 3′UTRs is distinct from that of the parent mRNAs [52].

One lncRNA that is expressed at a high level, and therefore may be effective as a ceRNA, is the previously-mentioned *H19*. This lncRNA is highly expressed in undifferentiated muscle cells, while its expression decreases in differentiated cells at about the same time as the expression of miRNA let-7 increases [53]. *H19* contains let-7 binding sites and siRNA-induced depletion of *H19* in mouse C2C12 muscle cells leads to reduced expression of let-7 target genes and increased expression of markers of muscle differentiation [53]. *H19* also binds to members of the miR-17-5p seed family. Expression levels of *H19* target mRNAs during myoblast differentiation suggest *H19* is competing for miR-17-5p binding [54]. Thus, *H19* acts as a primary transcript of one miRNA and a ceRNA for a number of others.

CircRNAs are potentially very stable because, unlike linear lncRNAs, they are resistant to exonuclease digestion. This feature raises the possibility that these RNA species may act as ceRNAs. The natural antisense transcript of the gene encoding cerebellar degeneration-related protein 1 (*CDR1-AS*, also known as *ciRS-7*) is one of the best-characterised circRNAs [55], [56], [57]. It is highly expressed in mouse hippocampus and neocortex, where expression of *CDR1-AS* overlaps with miR-7 expression domains [55], [57]. *CDR1-AS* contains a large number of miR-7 binding sites, which have been shown to deplete miR-7 and therefore to regulate expression of miR-7 target genes *in vitro* [55], [57]. Interestingly, there are mismatches in the central region of the miR-7 binding sites within *CDR1-AS* [55]. These render *CDR1-AS* resistant to miRNA-mediated degradation, thus the pool of ceRNA is not depleted by the process of competition, making it a very effective competitor [55]. In contrast, the binding site within *CDR1-AS* for miR-671 is nearly perfectly complementary, and so binding of this miRNA negatively regulates the level of *CDR1-AS* expression [55].

#### miRNA-independent mRNA degradation

In addition to regulating gene expression through an interaction with miRNA, there is evidence that some lncRNAs can directly target mRNA for degradation. Staufen 1 (STAU1) is a protein that recognises a specific motif in the 3′UTR of mRNAs and mediates their degradation by nonsense-mediated mRNA decay (NMD) [58]. For example, STAU1 binds to a double-stranded RNA motif within the 3′UTR of the mRNA encoding ADP-ribosylation factor 1 (*ARF1*) [59], where it is formed by a stem loop structure within the mRNA itself. However, some mRNAs targeted by Staufen-mediated decay, such as the one encoding serpin peptidase inhibitor, clade E member 1 (*SERPINE1*), contain only a single-stranded binding site within the 3′UTR, lacking the stem loop structure. Interestingly, it appears that such mRNAs may be targeted by a lncRNA carrying a complementary single-stranded binding site, imperfect binding of the lncRNA to the mRNA thus creating a double-stranded RNA binding motif for STAU1 [60]. This class of lncRNAs has been named half STAU1 binding site RNA (*1/2-SBS1RNA*) [60]. It is of note that the lncRNA terminal differentiation-induced ncRNA (*TINCR*) also recruits STAU1 to mRNAs such as the one encoding peptidoglycan recognition protein 3 (*PGLYRP3*) in epidermis [61]. Nonetheless, the interaction between *TINCR* and STAU1 does not trigger NMD and instead such binding increases stability of interacting mRNAs containing the TINCR box motif [61]. These findings suggest that a number of outcomes are possible, perhaps dependent on recruitment of additional factors.

### Transcriptional regulation by lncRNA

#### Transient lncRNA is transcribed from active enhancers

Transcription of most genes involves an interaction of a proximal promoter with more distant enhancer elements. Enhancers are located often at a large distance away from the transcriptional start site (TSS) and bind tissue-specific TFs that function to regulate differential gene expression [62]. The expression of a given gene is often regulated by the combinatorial effects of one or more enhancers, each active at a specific developmental time point or in a specific tissue. For example, expression of the gene *NODAL* during embryonic development is regulated by the interaction between at least five enhancers [63].

Active enhancers are bound by RNAPII, which may reflect their interaction with the promoter. By studying enhancers activated by calcium signalling in mouse neurons, Kim et al. [64] made the surprising discovery that an ncRNA of about 2 kb is bidirectionally transcribed from active enhancers. Expression of this enhancer RNA (eRNA) seems to be correlated with the activity of the enhancer [64], [65]. eRNA may either be polyadenylated [66] or lack polyadenylation [64], the latter suggesting transience and instability. However, a number of studies suggest that it may nevertheless be functional. For example, the nuclear receptors nardilysin 1 (NRD1) and NRD2 (also known as Rev-Erbs) regulate transcription of the target gene encoding matrix metallopeptidase 9 (*MMP9*) by inhibiting the expression of the eRNA transcribed from an *MMP9* enhancer [67]. Global run-on sequencing was used to characterise these transcripts, demonstrating that the *MMP9* enhancer is 983 bp in length and consists of a central core of 388 bp containing TF binding sites flanked by sequence encoding sense and antisense eRNAs [67].

It has been proposed that eRNAs may play a role in chromatin remodelling, acting to promote chromatin accessibility [66] and stabilise the DNA loop necessary to bring a distal enhancer into apposition with its promoter [68]. For instance, chromatin at the *FOXC1* locus is stabilised by a complex including the oestrogen receptor alpha (ERα) and its ligand, as well as an eRNA transcribed from a *FOXC1* enhancer [68].

#### Enhancer-like activity of lncRNA genes

**Figure 3:**
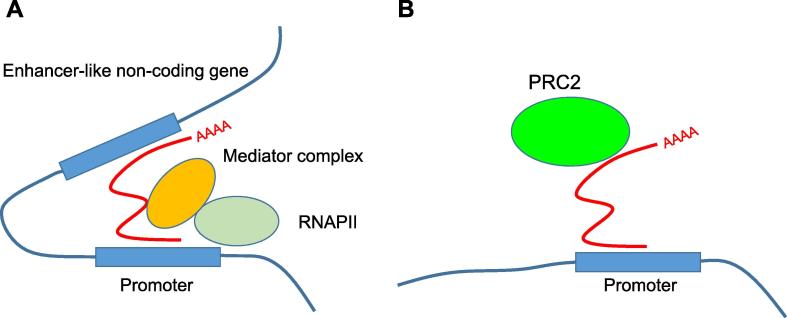
**Models of transcriptional regulation** In the bridging scaffold model (**A**), activating RNAs (red line) are transcribed from enhancer-like non-coding genes and are required to recruit the mediator complex and to mediate chromatin conformational changes bridging the enhancer-like non-coding gene and the promoter of a coding gene. In the tethered scaffold model (**B**), lncRNA (red line) recognises specific DNA motifs and recruits histone modifying enzymes such as PRC2 to the locus. lncRNA, long non-coding RNA; PRC2, polycomb repressive complex 2; RNAPII, RNA polymerase II.

A related class of lncRNAs is the activating ncRNAs (ncRNA-as). These are a species of lncRNAs transcribed from independent loci, but not from enhancers. They also have a transcriptional activation function [69], [70], [71]. ncRNA-as specifically activate the transcription of neighbouring coding genes in an RNA-dependent fashion, requiring the activity of the coding gene promoter [69]. Thus functionally they are highly similar to eRNAs. However, in contrast to eRNAs, ncRNA-as are spliced, polyadenylated stable transcripts. In common with both DNA enhancers and eRNAs, gene activation mediated by the ncRNA-a requires a change in chromosomal conformation to bring the ncRNA-a locus close to the promoter of its target gene [72]. Mediator is a protein complex which, along with cohesin, is involved in bridging together enhancers and promoters [73]. A number of ncRNA-as have been shown to be associated with the mediator complex, and depletion of the complex inhibits looping between the ncRNA-a locus and its target gene. Thus eRNA and ncRNA-a may function by interacting with the same set of molecules, forming a scaffold for a protein complex that bridges the enhancer-like element and the promoter of a coding gene (Figure 3).

While most circRNAs are cytoplasmic and thus may function in post-transcriptional regulation, a specific subclass of circRNAs demonstrates nuclear localisation and appears to function in transcriptional regulation. These circRNAs, in contrast to those described above, retain both exons and introns and therefore have been named exon–intron circRNAs (EIcircRNAs) [74]. EIcircRNAs are able to enhance transcription of their parental (coding) genes by interacting with RNAPII in an interaction that requires the U1 small nuclear RNA (snRNA) [74].

#### Transcriptional regulation by recruitment of chromatin modifiers

A number of the best-characterised lncRNAs, such as *X-chromosome inactivation* (*XIST*) and *H19*, are involved in processes such as X-chromosome inactivation (*XIST*) and imprinting (*H19*), in which a large region of a chromosome is inactivated such that only one copy is expressed [75]. In both cases, the lncRNA is expressed from the inactivated chromosome and has a binary relationship with its coding gene expression from the other chromosome, which is controlled by a *cis*-acting master control region [75]. *H19* is expressed exclusively from the maternally-inherited chromosome, while the adjacent gene encoding insulin-like growth factor 2 (*IGF2*) is expressed exclusively from the paternal chromosome [76], [77]. Deletion of *H19* in mice leads to maternal expression of *IGF2*, resulting in an overgrowth phenotype [78] which can be rescued by transgenic expression of *H19* [79]. Mechanistically, it has been shown that many of these lncRNAs are associated with chromatin modifying complexes and that this interaction directs inhibitory epigenetic modification of histones at adjacent loci. For example, the master control region lying between *H19* and *IGF2* consists of a region of differentially-methylated chromatin. It seems that *H19* is able to modify this control region by interacting with methyl CpG binding domain protein 1 (MBD1), which in turn interacts with histone lysine methyltransferases resulting in H3K9Me3 [80]. Another imprinted lncRNA, *antisense Igf2r RNA* (*AIR*), has been shown to be localised to the promoter of a target gene encoding solute carrier family 22 member 3 (*SLC22A3*) and to direct the histone methyltransferase G9a to this locus [81]. The imprinted *KCNQ1 overlapping transcript 1* (*KCNQ1OT1*) operates via a similar mechanism [15].

While many imprinted lncRNAs appear to act in *cis*, regulating nearby loci, there is also evidence that lncRNAs can act in *trans*. An elegant model for this is regulation of the homeobox (*HOX*) gene clusters, a group of homeotic genes that function to convey positional information in the embryo to establish the antero-posterior body axis. Each *HOX* gene is expressed in a specific domain along this axis, demonstrating colinearity, in which the order of genes on the chromosome reflects their expression domains in the body [82]. Remarkably, human fibroblasts retain this positional information in primary culture, permitting *in vitro* studies [83]. Differential gene expression is maintained by the creation of broad regions of open or closed chromatin within the *HOX* gene clusters by the action of the histone lysine methyltransferases polycomb repressive complex 2 (PRC2) and trithorax, which have opposing actions [84]. Over 200 lncRNAs are transcribed from the *HOX* clusters and seem to play a central role in the regulation of histone methylation states [84]. *HOX transcript antisense RNA* (*HOTAIR*), one of these lncRNAs expressed from the *HOX-C* cluster, has been shown to recruit PRC2 and lysine (K)-specific demethylase 1A (LSD1) to the *HOX-D* cluster by recognising a specific GA-rich DNA motif [85], maintaining repression over a 40-kb region of the chromosome [84], [86], [87]. Similarly, *XIST* also recruits PRC2 [88]. Indeed a genome-wide RNA immunoprecipitation experiment suggests that as many as 20% of lncRNAs across various human cell types may be associated with PRC2, suggesting that this is a general mechanism [89]. Another *HOX* cluster lncRNA, *HOX-A transcript at the distal tip* (*HOTTIP*), is transcribed from the 5′ end of the *HOX-A* cluster and targets a methylation complex to the locus required for expression of a number of *HOX-A* genes [90]. There are many more examples of this kind of interaction [23].

Details on the precise mechanisms underlying these activities are unclear. It seems that the lncRNA must serve two functions: (1) it must bind to a protein or protein complex, or at least facilitate the formation of a complex, perhaps acting as a scaffold [87]; and (2) the lncRNA must be able to target this complex to a specific DNA sequence. Thus, the function of the lncRNA is to provide specificity to the chromatin modifying enzymes, acting as a tethered scaffold (Figure 3).

Histone modifications have been demonstrated to be able to influence alternative splicing of mRNA, acting through an adaptor complex consisting of a chromatin-reading protein linked to RNAPII and the splicing machinery [91]. For example, a different splice form of the gene encoding fibroblast growth factor receptor 2 (*FGFR2*) is expressed in epithelial cells compared to that expressed in mesenchymal cells. It has been shown that differential splicing is dependent on the methylation state of histone H3K36 and H3K27 at the *FGFR2* locus [91]. An antisense lncRNA to *FGFR2*, *asFGFR2*, is expressed in epithelial cells and acts to repress mesenchymal-type splicing by recruiting PRC2 and the histone demethylase lysine-specific demethylase 2a (KDM2a) to the locus [92].

## Concluding remarks

In this review we have described the highly diverse biological functions of lncRNAs, reflecting the versatility of the RNA molecule itself. We have proposed a general model in which these functions may broadly be divided into those representing an interaction with miRNA networks in order to regulate gene expression at the post-transcriptional level and those representing an interaction of lncRNA with enhancers, promoters, and chromatin-modifying complexes to regulate gene expression at the transcriptional level. Some lncRNAs such as *H19* appear to act at multiple levels of gene regulation. It remains to be seen whether this is a general phenomenon. Indeed, it should be noted that much of our current understanding of lncRNA comes from studies of a small number of lncRNAs and it is presently unclear whether these are representative of the group as a whole. One thing that seems likely is that as we begin to understand these molecules better, the rather crude classification into long and short ncRNAs will need to be refined to better reflect their diverse functions.

Any review on this subject will be always incomplete for the simple reason that the field is rapidly expanding. In addition to the mechanisms described here, lncRNA has also been implicated in the regulation of mRNA splicing by modulating the levels of serine/arginine splicing factors within nuclear speckles [93]. An antisense lncRNA overlapping the start codon of ubiquitin carboxy-terminal hydrolase L1 (*UCHL1*) has been shown to positively regulate translation [94]. These lncRNAs consist of two domains: one domain overlapping the coding gene, which confers target specificity, and the other domain containing a SINEB2 repeat element that seems to recruit polysomes [94]. In contrast, *lincRNA-p21* inhibits translation by recruiting the translational repressor Rck [95]. Finally, the lncRNA *growth arrest-specific 5* (*GAS5*) has been shown to act as a DNA mimic, sequestering the glucocorticoid receptor, and acting as a decoy to prevent its binding to a DNA motif in target genes [96] in an interesting parallel to the sequestration of miRNAs described above.

While we have made great strides in our understanding of lncRNA, we are still only at the start of this road. Many challenges must be overcome. One difficulty facing the field is the low level of cross-species conservation of the many lncRNAs, which makes the use of model organisms such as mice or fish difficult. This creates challenges to the understanding of lncRNA functions in whole body processes such as embryonic development and complex diseases. This lack of primary sequence conservation could be interpreted to mean a lack of evolutionary constraints on lncRNA. However, some have argued that we should not apply the same criteria for sequence conservation to lncRNA as we do to coding genes because lack of sequence conservation does not in itself indicate lack of functional conservation [97] and evolution may act on lncRNA at another level [98]. For example, the lncRNA *HOTAIR* shows poor primary sequence conservation between humans and mice, yet both its function and its genomic location within the *HOX-C* cluster are conserved [86].

In fact, there is some evidence that selection may act on lncRNA at the structural level rather than at the level of the primary sequence [99], [100]. This raises the possibility that functional orthologues may be studied in model organisms. For this reason, methods to determine the structure of lncRNA will become increasingly important. Traditionally RNA secondary structures have been determined using either nuclease digestion, in which enzymes specifically cleave either single or double stranded RNA, or with the use of chemicals to modify exposed nucleotides [101]. Such methods have been successful in determining the structure of individual lncRNAs such as *HOTAIR* [102]. However we need to find ways to adapt these methods for high-throughput genome-wide applications. Early attempts to do just this include parallel analysis of RNA structure (PARS) [103] and FraqSeq [104], in which RNA sequencing is used to analyse the resulting products of nuclease digestion.

Another important line for future research will be to determine the binding partners of lncRNA. Many studies take a protein-centric approach to this problem and use an antibody to purify all lncRNAs associated with a particular protein. This is useful in identifying that many lncRNAs associate with the PRC2 complex [89]. However, in some cases it is more helpful to look at this problem from an RNA-centric viewpoint. A useful tool for such studies is the S1 tag, an RNA motif that mimics the structure of biotin and may thus be purified from cell extracts using streptavidin [105], [106]. The S1 tag is short and so may be used to tag lncRNA molecules in a similar manner to protein tags such as HA and V5. This method has been successfully employed to identify binding partners of the lncRNA *H19* [53].

One of the exciting prospects arising from our increased understanding of the lncRNA field is the possibility of understanding the roles of lnRNA in human disease. GWAS analyses have in the past tended to focus only on the coding part of the genome, although it has been known for some time that certain diseases show a strong association with regions of the genome lacking coding genes. For example, multiple SNPs linked to a number of cancers map to a gene desert surrounding the oncogene *MYC* at the 8q24 locus [107]. As it becomes clear that lncRNA is both abundant within the genome and that it has a distinct biological role, it is logical to suggest that mutations within these sequences will be found to have clinical implications. Indeed this seems to be the case and a number of lncRNAs are now known to be transcribed from 8q24 and to regulate the expression of *MYC* [108]. Similarly in heart disease, SNPs mapped to a number of lncRNAs including *myocardial infarction associated transcript* (*MIAT*) [109], *antisense non-coding RNA in the INK4 locus* (*ANRIL*) [110], and the aforementioned *H19* [111] have been shown to have a disease association. The challenge facing such research will be to predict which of the many variants within the population are functionally significant, a question that can only be answered by functional studies such as those described in this review.

## Competing interests

The authors declare that there are no conflicts of interests.
